# Is Euro-Collins better than ringer lactate in live related donor renal transplantation?

**DOI:** 10.4103/0970-1591.33722

**Published:** 2007

**Authors:** G. Siva Prasad, Chacko N. Ninan, Antony Devasia, Lionel Gnanaraj, Nitin S. Kekre, Ganesh Gopalakrishnan

**Affiliations:** Department of Urology, Christian Medical College, Vellore - 632 004, India

**Keywords:** Euro-Collins, live donor renal transplantation, perfusion fluid, ringer lactate

## Abstract

**Objectives:**

Euro-Collins and University of Wisconsin are preferred solutions in cadaveric renal transplantation. There are no guidelines regarding the perfusion fluids in live donor renal transplantation. We studied whether Euro-Collins was better than Ringer lactate in terms of protecting allograft function.

**Materials and Methods:**

A double-blind permuted randomized trial comparing Euro-Collins and Ringer lactate was performed on 100 patients undergoing live related donor renal transplantation. Outcome variable was serum creatinine.

**Results:**

Age, sex, donor nephrectomy and ischemia times, kidney temperature, time of first appearance of urine was not significantly different in both the groups. Fall in serum creatinine was significantly more in Euro-Collins than Ringer lactate in the first postoperative week (*P*-<0.05). The time to reach nadir creatinine was 4.97 days in Euro-Collins and 7.75 days in the Ringer lactate group (*P*-0.088). Serum creatinine was significantly lower in the Euro-Collins group till six months, thereafter it equalized with Ringer lactate. When individual parameters were analyzed for time to nadir creatinine, only the cold ischemia time of > 80 min was found to be significant (*P*-0.024). Twelve kidneys in Euro-Collins and 17 in the Ringer lactate group had cold ischemia times of ≥80 min and time to nadir creatinine was 4.33 ±3.74 and 12.76± 12.68 days (*P*-0.035).

**Conclusions:**

Renal function normalized rapidly when Euro-Collins was used. Cold ischemia time of ≥ 80 min was the most important factor affecting the graft function and perfusing with Euro-Collins could protect the allograft.

Early graft function is the primary aim of any transplant program. It significantly relates to long-term outcome.[1] At present, 20-25%[2] of kidney transplants in the world are with living donors, whereas in India it still forms >90% of the donor pool. Preservation of the viability of the graft between explantation and implantation is vital for successful transplantation. Simple hypothermia is not enough for preserving the viability of the graft. Cooling diminishes metabolic activity and curtails the oxygen demand of the preserved organ. Metabolism still continues. Oxygen consumption decreases at an exponential rate with fall of temperature to 10% of the normal at 10°C.[3] Cellular homeostasis is not preserved during hypothermia. Active transport mechanism involving sodium, potassium and calcium, magnesium ATPase is inhibited at < 10°C, but passive diffusion of these ions occurs.[4] There is increased accumulation of lactate from anaerobic glycolysis. This lowers pH and causes instability of lysosomal enzymes and generation of free radicals. Membranous lipid fluidity is diminished causing cellular swelling and cell death.

Various organ preservation solutions (Citrate, Collins, buffered sucrose, Bretschneider, University of Wisconsin and Celsier) have evolved over the last 50 years. Various centers use different fluids for perfusion. At our center, we have been using cooled Ringer lactate solution with heparin and papaverine along with surface cooling with ice slush in living donor renal transplantation for the last 30 years. After live donor nephrectomy the kidney cooled using preservation solutions soon after extraction, hence it is believed that any of the preservation fluids can be used for perfusion.[4] There are no randomized controlled trials to support the evidence that one solution is better than the other in living donor transplantation. It was hypothesized that when transit time between the nephrectomy and implantation was minimal, only cooling of the kidney would suffice, but intracellular electrolyte imbalance was not accounted. There are situations where the renal revascularization time could be prolonged and this cannot be predicted. In such situations, the perfusion fluid that has an intracellular fluid composition would be able to maintain cellular homeostasis better than Ringer lactate. Our aim was to study whether the perfusion fluids’ composition affects the graft function in terms of time to nadir creatinine and serum creatinine.

## MATERIALS AND METHODS

End stage renal disease patients, who underwent first live related donor renal transplantation at our center were considered for the study. Institutional ethical review board and informed consent was obtained. All 100 consecutive patients consented for the study. They were randomized by permuted block randomization to receive one of the cooled perfusion fluids. The study was conducted from February 2003 to February 2004. Both donor and recipient surgeries were performed by various surgeons in the Urology department at out center. Donor nephrectomy was performed either by open or laparoscopic method. Intraoperative fluid management is well standardized at our center and is confirmed by noting a full renal artery with no spasm, an even perfusion of the kidney and the presence of urine output on disconnection of the ureter. The Perfusionist (investigator), patient and statistician were blinded to the type of fluid used. Perfusion fluids used were either Ringer lactate (RL) or Euro-Collins (EC) [Table 1]. Both fluids were cooled to a fixed temperature (4-8° C) in the refrigerator overnight (temperature gauge check was used). Kidneys were perfused by a non-pulsatile continuous method using one liter of one of the fluids. Thermometer based on thermocouple principle was used to measure kidney temperature, by placing the blunt atraumatic thermometer probe into the renal vein at the end of the perfusion. The following data was recorded:

- Age / Sex (donor and recipient).
- Donor Relationship with the recipient.
- HLA Matching.
- Donor nephrectomy time (DNT) -Time period between the skin incision and clamping of renal artery.
- Warm ischemia time (WIT) -Time period from the clamping of renal artery to the complete clearance of blood from the renal vein during perfusion.
- Kidney temperature (KT) – The thermometer was placed in the renal vein for 30sec to achieve a steady state before recording the temperature.
- Perfusion time (PT).
- Cold ischemia time (CIT) - Time period between the complete clearance of blood from the renal vein during perfusion to release of the recipient's vascular clamp after anastamosis.
- Time taken for first appearance of urine after renal revascularization (UO1).
- Persistent hypotension in the recipient after revascularization requiring inotropic support (Systolic BP < 90mmHg).
- Central venous pressure at the clamp release (CVP).
- Intraoperative technical problems.
- Serum creatinine level (mg/dl) on postoperative Days

**Table:** 

0	1	2	3	4	5	6
7	14	30	90	180	360	

(Day 0 being the day of transplantation).
- Nadir serum creatinine – Time taken to reach lowest possible serum creatinine.
- Delayed graft function (DGF) - defined as a need for dialysis in the post-transplant period or serum creatinine value failing to decrease spontaneously.

**Table 1:** Perfusion fluid composition

Euro-Collins (EC) 1000 ml	Ringer lactate (RL) 1000 ml
Glucose anhydrous IP 35.7g	Calcium chloride IP 0.27g
Monobasic potassium phosphate	Potassium chloride I.P 0.40g
USP-NF 2.04g	Sodium chloride IP 6.0g
Dibasic potassium phosphate USP 7.40g	Sodium lactate IP 3.2g
Potassium chloride IP 1.12g	
Sodium bicarbonate IP 0.84g	
Osmolarity 529 mOsm/l	
K^+^: 115 mmol/l, Na^+^: 10 mmol/l,	
cl^−^: 15 mmol/l, HCO_3_: 10 mmol/l,	
H_2_Po_4_: 15 mmol/l, HPo_4_: 42.5 mmol/l	
pH: 7.25(4°C)	

Early graft function was measured by the fall in the serum creatinine in the postoperative period and by time to reach nadir level of serum creatinine. Graft function was assessed by the serum creatinine at three, six months and one year. Subgroup analysis with high-risk factors like elderly donors>50 years, prolonged ischemia time and multiple vessels was done. Sample size was determined by calculating the differential mean and standard deviation of the drop in serum creatinine level in 35 consecutive living donor renal transplant patients selected randomly. Statistical analysis was done by linear regression analysis, compared means with independent-samples T test and descriptive variables by Pearson Chi-square test and Fisher's exact test.

## RESULTS

Ninety-nine patients out of 100 studied were analyzed, 50 in the Euro-Collins group (EC) and 49 in Ringer lactate group (RL). One patient in RL was not considered for analysis, as the data collection was incomplete. All baseline parameters were comparable in both the groups [Table 2]. Youngest donor was 21 years old and oldest 61 years. Distribution of laparoscopic and open donor nephrectomy was also equal among the groups. Two patients in the RL group had persistent hypotension after release of clamps and required inotropic support, one of them had prolonged cold ischemia time >80min and had delayed graft function. All patients received cyclosporin-based immunosupression except two in the RL group and subgroup analysis for time to nadir creatinine was not done.

**Table 2:** Baseline data

Parameter	Euro-Collins	Ringer lactate	*P*-value
Donor's age (yrs)	44±10	43±10	0.487
Recipient's age (yrs)	33±12	36±13	0.311
Sex (M/F)	37/13	43/6	0.082
DNT	163 ± 36 min	189 ± 39 min	0.148
Warm ischemia time	3 ± 1	3 ± 1	0.442
Perfusion time	18 ± 3	18 ± 4.6 min	0.633
Kidney temperature	10 ± 1.90	10 ± 2.00	0.803
Cold ischemia time	71 ± 17 min	73 ± 20 min	0.599
UO1	10.1 ± 33.8 min	9.0 ± 19.2 min	0.880
Hypotension	0	2	0.152
CVP	11.9 ± 3.03 mmHg	11 ± 3.09 mmHg	0.354
Multiple arteries	3	5	0.487

**Figure 1:**
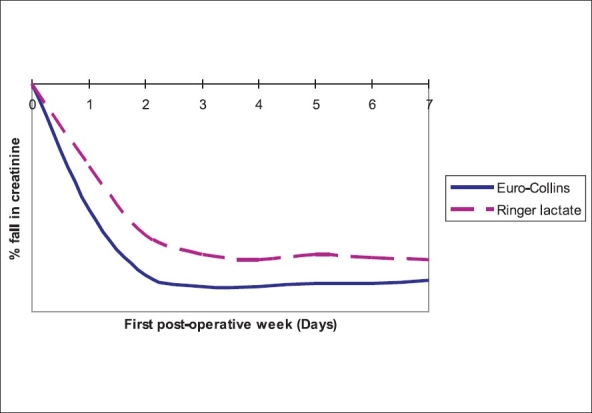
Rate of serum creatinine fall

**Figure 2:**
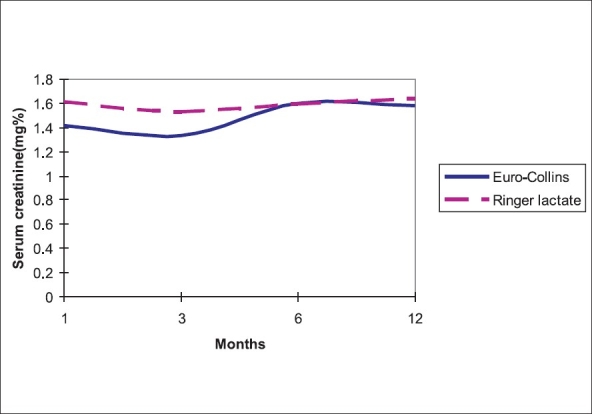
Allograft function in first year

The percentage fall of serum creatinine in the first postoperative week was significantly more in the EC than RL group [Figure 1]. Allograft function, measured by the levels of serum creatinine attained was significantly better in the first six months in the EC group compared to the RL group [Figure 2], however, this difference nullifies after six months. The time to reach nadir level of serum creatinine was 4.97 days (SD+ 6.31) in the EC group and 7.75 days (SD + 9.34) in the RL group. The difference was not statistically significant (*P*-0.088). Eleven patients had delayed graft function (DGF) by definition, three in EC and eight in RL group. Serum creatinine at three months in DGF patients was not different from the other non-DGF group (1.40 +0.40 Vs1.58 +0.36, *P*-0.181).

**Figure 3:**
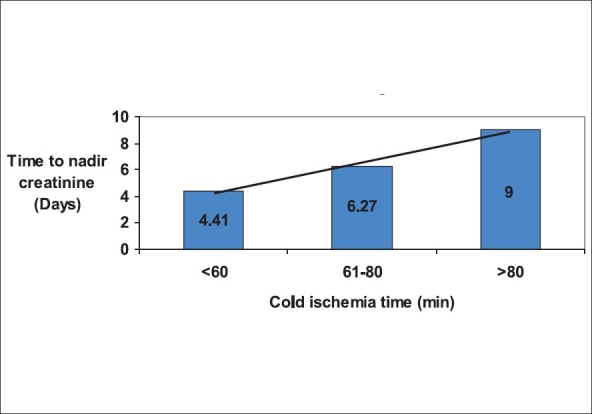
Effect of cold ischemia time on allograft function

We considered age > 50 years as elderly donors, they were 33, 18 in the EC and 15 in the RL group. In 25 patients the DNT was ≥ 3h; WIT ≥ 5 min was present in 14 patients. Eight had multiple vessel anastamosis. When these high-risk factors, including cold ischemia time were studied by linear regression analysis with dependent variable as time to nadir creatinine, only cold ischemia time was found to be significant. Time to reach nadir serum creatinine was significantly affected when the cold ischemia time (CIT) was ≥ 80 min [Figure 3].

**Figure 4:**
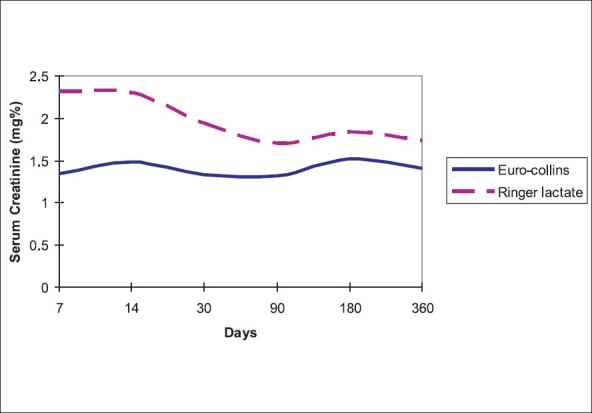
Allograft function among CIT > 80 min

Twelve patients in the EC and 17 in the RL group had CIT ≥80 min and time to reach nadir serum creatinine was 4.33 ± 3.74 and 12.76 ± 12.68 days. The difference was statistically significant (*P*-0.035). The graft function in patients with CIT ≥ 80min was significantly better in the EC group compared to the RL group [Figure 4]. Mean CIT among patients with multiple artery anastamosis was 77 min 25 sec compared to 72 min 9 sec in those with single artery anastamosis. In our study CIT was not significantly increased by multiple vessel anastamosis.

## DISCUSSION

Live related donor renal transplantation has better outcome than cadaveric transplantation.[5] The main factor being a short CIT. It is often believed that when transit time (i.e. time from removal of the kidney to implantation) is short, simple cooling of the kidney is enough for preserving the organ. Many centers use fluids with extracellular fluid composition for renal perfusion in live donor transplantation. Cellular metabolism proceeds at a slower rate even during hypothermia[6] and intracellular homeostasis is not maintained. Hence there is inevitable intracellular ionic disturbance and nutritional depletion, which could result in delayed graft function. Delayed graft function (DGF) after live related donor transplantation affects 5-10% of recipients.[7] This delay in function is associated with an increased risk of rejection and decreased graft survival.[7] Delayed graft function was 11% in our study and it did not affect graft function at three months. The fall in serum creatinine was significantly higher on the first and second postoperative days among kidneys perfused with EC. However, time taken to reach nadir serum creatinine level was not significantly different. It appears that renal allograft reverts back to near normal function earlier, if it is perfused with an intracellular fluid composition (Euro-Collins). The serum creatinine levels attained in the first six months after transplantation were significantly better in kidneys perfused with Euro-Collins [Figure 1]. There are no randomized studies done on humans comparing perfusion fluids with intracellular and extracellular composition on the outcome of live related donor renal transplantation. To assess the immediate renal allograft function in live donor transplantation, Bugge JF[8] compared transplanted kidneys with corresponding live donor kidneys in eight patients. He observed that renal allograft function tended to normalize within 24h of implantation. He used Euro-Collins for perfusion. In canine kidney preservation model[9,10] renal function was found to be better in kidneys perfused and preserved with solutions with intracellular composition than extracellular composition. However, there are conflicting results showing homogenous cooling and flushing was more important than the composition in another canine kidney preservation model.[11]

GFR declines with aging.[12] There in a fall of GFR by 25% at 50 years and it declines thereafter by 10% each decade. Donor's age is an important factor in the outcome of allograft function. There are more chances of DGF if the donor is ≥ 50 years and the graft survival rate is lesser by 14-25% at one, five and 10 years.[13] In our study, kidneys from donors over 50 (33%), had no significant difference in graft function when compared to younger donors. Longer DNT was shown to have debatable increased incidence of DGF.[14,15] We had 14 patients who had DNT lasting for ≥ 3h. Graft function was not affected in them. When there is an adequate intraoperative fluid management and proper handling of the kidney, the cellular homeostasis is maintained, hence prolonged DNT does not appear to affect graft function.

Studies comparing open nephrectomy and hand-assisted laparoscopic nephrectomy have emphasized on decreased WIT when compared with pure laparoscopic retrieval. No data exists that defines exactly what constitutes a prolonged WIT in terms of recipient graft function. Up to 10 min of warm ischemia was not found to be significant.[16] Mean WIT in our study was 3 min in both the groups. There were 14 patients in whom WIT was ≥5 min. Graft function was not affected in this group. Recommended kidney temperature (KT) for hypothermia is 8-18°C.[4] In our study the mean KT was 10°C in both the groups. There was no effect on graft function when the temperature was ≥10°C. Kidney temperature increases according to the logarithmic curve given by T=7.2 ln (t)-0.6 during revascularization.[17] It is desirable that vascular anastamosis time be reduced and KT is sufficiently low to account for kidney warming up during revascularization.

Early postoperative urine flow was found to predict DGF in cadaveric renal transplantation.[18] In our study, time of first appearance of urine after revascularization (UO1) was not significantly different in both the groups. Patients with DGF did not have statistically significant delay in urine output. The diuretic used in DGF patients could have confounded this. According to several autopsy series, the incidence of multiple renal arteries ranges between 18 and 30%. Contrary to the belief that multiple vessels affect graft function, there was no significant difference found in graft and patient survival between multiple and single renal artery allograft in some studies.[19] In our study, there were eight patients with multiple arteries and this had no impact on graft function.

Cold ischemia time was the most important factor in the outcome of renal transplantation. Shorter the CIT the better is graft function. Prolonged ischemia has been linked to DGF.[1] Our own unpublished retrospective data showed that DGF was likely to occur when the CIT was ≥90 min. Delayed graft function directly correlates with the long-term outcome of the graft. In our study, a CIT of ≥80 min affected the allograft function significantly and the time to reach nadir creatinine level was three times more when the kidneys were perfused by RL. We could have prevented this delay in onset of graft function if we had used EC for perfusion. Further, among patients with CIT>80 min the graft function itself was much better over one year. Although we cannot draw a direct correlation between the perfusion fluid composition and the allograft function, we can hypothesize that perfusion fluids with intracellular composition can maintain cellular homeostasis during prolonged cold ischemia, facilitate early graft function and provide better outcome during the first six months. However, we cannot explain why this effect nullifies after six months.

## CONCLUSIONS

Use of perfusion fluids with intracellular composition allows the graft function to normalize sooner. Cold ischemia time of more than 80 min affects graft function significantly. Perfusion with Euro-Collins can protect the graft function in patients with longer cold ischemia.
